# Connector Design and Pontic Span Length as Determinants of Mechanical Performance in Zirconia Fixed Dental Prostheses: A Systematic Review

**DOI:** 10.3390/dj14080500

**Published:** 2026-08-08

**Authors:** Andrea Ordoñez Balladares, Luis Chauca-Bajaña, Dario Adolfi, Míriam Teulé-Trull, Tiago Reis, José Martín-Cruces

**Affiliations:** 1 College Dentistry, University Bolivariana del Ecuador, Durán 092406, Ecuador; andrea.ordonezb@ug.edu.ec (A.O.B.); jose.martin.cruces@usc.es (J.M.-C.); 2 School of Dentistry, Universidad Católica de Santiago de Guayaquil (UCSG), Guayas 090101, Ecuador; 3 Private Practice, São Paulo 04543-000, SP, Brazil; darioadolfi@gmail.com; 4 Department of Surgery and Medical-Surgical Specialties, School of Medicine and Dentistry, University of Santiago de Compostela, 15701 Santiago de Compostela, Spain; miriamteule@gmail.com; 5 Department of Endodontics, Faculty of Dentistry, Universitat Internacional de Catalunya, 08195 Barcelona, Spain; 6 FP-I3ID, FP-BHS, Faculty of Health Sciences, University Fernando Pessoa, 4200-150 Porto, Portugal; tiagofaria@ufp.edu.pt; 7 CDRSP, Polytechnic University of Leiria, 2430-028 Marinha Grande, Portugal

**Keywords:** zirconia, fixed dental prosthesis, connector design, pontic span length, fracture resistance, finite element analysis

## Abstract

**Objectives**: To systematically evaluate the influence of connector design and pontic span length on the mechanical performance of zirconia fixed dental prostheses (FDPs). **Methods**: A systematic review was conducted according to PRISMA 2020 guidelines (8). Electronic searches were performed in PubMed, Scopus, Web of Science, and Embase. In vitro and finite element analysis (FEA) studies evaluating zirconia FDPs were included. **Results**: A total of 11 studies were included. Across all studies, connector regions were consistently identified as the primary site of fracture initiation. Reduced connector dimensions significantly decreased fracture resistance and fatigue life, whereas increased cross-sectional area and optimized geometry improved stress distribution and mechanical performance. Longer pontic spans were associated with increased stress concentration and reduced structural stability. **Conclusions**: Connector design and pontic span length are critical determinants of the biomechanical behavior of zirconia FDPs. Clinical significance: Optimization of connector dimensions and geometry is essential to improve the longevity and reliability of zirconia FDPs.

## 1. Introduction

Zirconia-based fixed dental prostheses (FDPs) currently occupy a prominent position in prosthodontic rehabilitation owing to their favorable combination of mechanical strength, biocompatibility, chemical stability, and esthetic properties. Since the introduction of yttria-stabilized tetragonal zirconia polycrystal (Y-TZP), continuous advancements in its chemical composition, microstructure, and manufacturing processes have led to the development of materials with enhanced optical properties and a more natural appearance, substantially expanding their clinical applications. In parallel, the incorporation of CAD/CAM technologies has enabled higher levels of precision and reproducibility in restoration fabrication, contributing to the consolidation of zirconia as one of the benchmark restorative materials in contemporary dentistry [1,2].

The high survival rates reported for both tooth-supported and implant-supported restorations have reinforced the clinical predictability of zirconia FDPs [3,4]. Nevertheless, progressive improvements in materials and manufacturing techniques have not completely eliminated the occurrence of mechanical complications. Framework fractures, connector failures, and loss of structural integrity continue to be reported in the literature, even in restorations fabricated from next-generation zirconia materials [5,6]. This issue is particularly relevant in multi-unit restorations, where functional demands are greater and the clinical consequences of mechanical failure are often more complex. Consequently, research interest has gradually shifted from the investigation of the intrinsic properties of zirconia toward the identification of structural factors that influence restoration performance under clinical loading conditions.

Although zirconia exhibits superior mechanical properties compared with many ceramic materials used in dentistry, the longevity of an FDP largely depends on how functional loads are distributed throughout the prosthetic structure. Findings from fractographic studies, in vitro investigations, and FEA consistently demonstrate that fractures do not occur randomly but tend to originate in specific regions subjected to critical stress concentrations. Among these regions, the connector area has repeatedly been identified as the most vulnerable component of the restoration, acting as the primary site for crack initiation and subsequent structural failure of the prosthesis [5,6]. This observation explains why connector design remains one of the most extensively investigated aspects of zirconia FDP biomechanics.

The influence of connector dimensions on restoration strength has been widely documented. In general, reductions in connector height, width, or cross-sectional area are associated with decreased load-bearing capacity and fatigue resistance, whereas larger dimensions promote a more favorable stress distribution and improved structural stability [7]. However, translating these findings into clinical practice is not always straightforward. Anatomical limitations, available restorative space, and esthetic requirements frequently constrain design possibilities, necessitating compromises between biomechanical principles and clinical demands. As a result, uncertainty persists regarding the minimum connector dimensions required to ensure predictable mechanical performance without adversely affecting other treatment objectives.

Connector dimensions represent only one aspect of the problem. The geometric configuration of this region also plays a critical role in determining stress distribution within the prosthetic framework. Abrupt transitions, sharp internal angles, and small curvature radii may promote localized stress concentrations, thereby increasing the risk of crack initiation. In contrast, designs with smoother and more gradual transitions tend to provide a more homogeneous load distribution and improved fracture resistance [8]. These findings indicate that the structural performance of an FDP depends not only on the amount of material present in the connector region, but also on how this material is integrated into the overall restoration design.

Pontic span length is another key factor influencing the mechanical behavior of zirconia FDPs. An increased distance between abutment teeth alters loading conditions by generating higher bending moments and greater stress concentrations within the prosthetic framework. As a result, long-span restorations are more prone to deformation and mechanical failure than shorter-span prostheses. This consideration is especially relevant in extensive rehabilitations, where functional demands are often increased. Moreover, available evidence suggests that the influence of span length is amplified when connector dimensions are reduced, underscoring the close interaction between these two variables [9]. From a biomechanical perspective, it is unrealistic to evaluate connector design and pontic span length as entirely independent factors. Modifications in either parameter affect the stress distribution throughout the entire prosthetic structure and may substantially alter its response to functional loading. Therefore, a configuration that performs adequately in a short-span restoration may not provide the same level of reliability when applied to a longer-span FDP.

Although the number of studies investigating the biomechanical behavior of zirconia FDPs has increased considerably over the past decade, the translation of these findings into clinically applicable recommendations remains limited. Significant heterogeneity exists among studies, particularly regarding prosthetic configurations, connector designs, artificial aging protocols, and methodologies used to assess mechanical performance, making direct comparisons difficult. Furthermore, experimental investigations and FEA provide complementary perspectives that do not always lead to equivalent conclusions. This situation has contributed to the persistence of uncertainty regarding the magnitude of the influence exerted by connector design and pontic span length on the structural reliability of zirconia FDPs [10].

In this context, a critical synthesis of the available evidence is necessary to clarify the true impact of these variables on fracture resistance, stress distribution, and the mechanical longevity of zirconia restorations. A deeper understanding of these factors may contribute to the establishment of more predictable and clinically applicable design criteria for the planning of multi-unit fixed dental prostheses. Therefore, the aim of the present systematic review was to evaluate the influence of connector design and pontic span length on the biomechanical performance of zirconia fixed dental prostheses [10].

## 2. Materials and Methods

### 2.1. Protocol and Reporting

**Figure 1:**
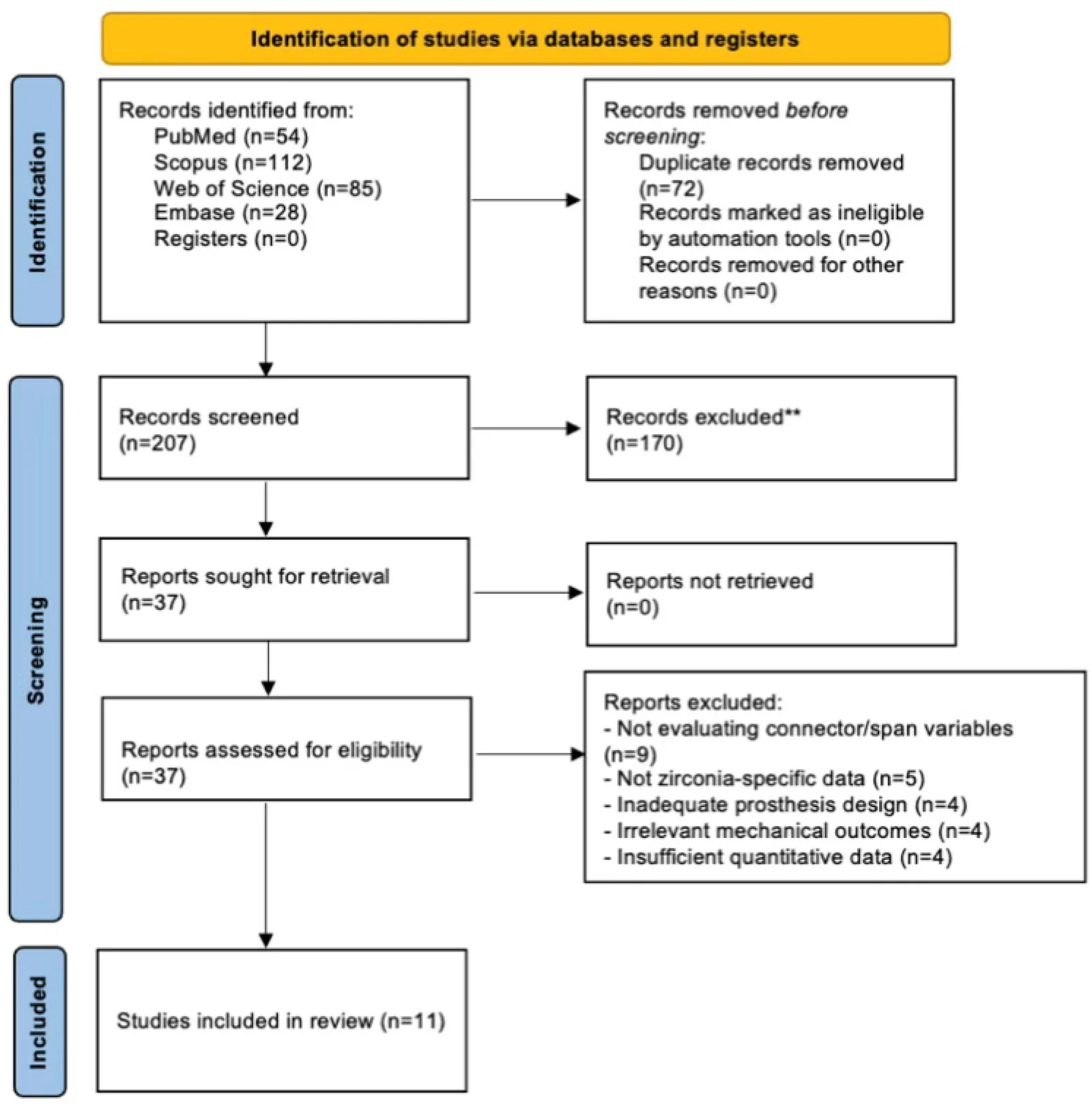
PRISMA flowchart. ** Records excluded after title and abstract screening because they did not meet the predefined inclusion criteria.

The PROSPERO database was searched in May 2025 to identify existing or ongoing systematic reviews on the topic. The protocol for the present systematic review was prospectively registered (registration number: CRD420261362019). This study was conducted and reported in accordance with the Preferred Reporting Items for Systematic Reviews and Meta-Analyses (PRISMA 2020) guidelines, and the study selection process is illustrated in the PRISMA flow diagram (Figure 1). In addition, a completed PRISMA 2020 Checklist is provided as Supplementary Material (File S1) [11].

### 2.2. PICO Question

Based on this framework, the focused research question was: How do connector design and pontic span length influence the mechanical performance of zirconia-based fixed dental prostheses?

The research question of this systematic review was formulated according to the PICO framework as follows: Population (P): zirconia-based fixed dental prostheses (FDPs) evaluated in in vitro and FEA studies; Intervention (I): variations in connector design, including cross-sectional area, height, width, and geometry; Comparison (C): different connector configurations and/or variations in pontic span length; Outcome (O): mechanical performance outcomes, including fracture resistance, fracture load, stress distribution, fatigue behavior, and failure mode.

### 2.3. Search Strategy and Database Screening

The Rayyan QCRI platform (Qatar Computing Research Institute, Doha, Qatar) was used for the identification, organization, duplicate removal, and screening of eligible studies. A comprehensive electronic literature search was performed in PubMed, Embase, Scopus, and Web of Science from database inception to 30 January 2026. No restrictions regarding publication year were applied. The search was limited to studies published in English and available as full-text articles.

The search strategy was tailored for each database using a combination of Medical Subject Headings (MeSH), Emtree terms, and free-text terms related to zirconia fixed dental prostheses, connector design, pontic span length, and mechanical performance. The main search concepts included “zirconia”, “fixed dental prosthesis”, “fixed partial denture”, “dental bridge”, “connector design”, “connector cross-sectional area”, “connector geometry”, “connector height”, “connector width”, “pontic span length”, “fracture resistance”, “fracture load”, “stress distribution”, “fatigue resistance”, and “finite element analysis”. Boolean operators (AND/OR) were used to combine terms and optimize search sensitivity.

The PubMed search strategy was as follows: ((“zirconia”[Title/Abstract] OR “zirconia-based”[Title/Abstract] OR “yttria-stabilized tetragonal zirconia”[Title/Abstract]) AND (“fixed dental prosthesis”[Title/Abstract] OR “fixed partial denture”[Title/Abstract] OR “dental bridge”[Title/Abstract]) AND (“connector design”[Title/Abstract] OR “connector cross-sectional area”[Title/Abstract] OR “connector geometry”[Title/Abstract] OR “connector height”[Title/Abstract] OR “connector width”[Title/Abstract] OR “pontic span length”[Title/Abstract]) AND (“fracture resistance”[Title/Abstract] OR “fracture load”[Title/Abstract] OR “stress distribution”[Title/Abstract] OR “fatigue resistance”[Title/Abstract] OR “finite element analysis”[Title/Abstract])).

The electronic search was supplemented by manual screening of the reference lists of all included studies and relevant peer-reviewed articles to identify additional eligible records not captured by the database search. Grey literature was included because the review was restricted to peer-reviewed full-text in vitro and finite element analysis studies. All retrieved records were imported into Rayyan QCRI, where duplicates were removed prior to screening. Study selection was subsequently conducted in two stages: title and abstract screening, followed by full-text assessment based on the predefined eligibility criteria.

### 2.4. Eligibility Criteria

Inclusion criteria:

- Study design: In vitro experimental studies and FEA studies published as full-text articles in peer-reviewed journals.
- Population/material: Zirconia-based fixed dental prostheses (FDPs), including multi-unit restorations such as 2-unit and 3-unit FDPs.
- Intervention/exposure: Studies evaluating connector design variables, including cross-sectional area, height, width, geometry, or curvature, and/or pontic span length.
- Comparator: Different connector configurations and/or variations in pontic span length within zirconia FDPs.
- Outcomes: Mechanical performance outcomes, including fracture resistance, fracture load, stress distribution, fatigue behavior, deformation/displacement, and failure mode.
- Language and availability: Studies published in English with full-text availability.

Exclusion criteria:

- Study design: Clinical studies, case reports, case series, reviews, systematic reviews, meta-analyses, editorials, letters, and conference abstracts.
- Population/material: Studies evaluating single crowns or restorations not classified as fixed dental prostheses, as well as studies on materials other than zirconia without separate data.
- Intervention/exposure: Studies not specifically analyzing connector design or pontic span length as primary variables.
- Outcomes: Studies not reporting relevant mechanical outcomes or lacking sufficient data for qualitative analysis.
- Language and availability: Studies not published in English or without full-text access.

### 2.5. Studies Screening and Data Extraction

An ad hoc standardized data extraction sheet was developed and completed independently by three investigators (LC, AOB, and JMC) using a customized data collection form. Any disagreements or uncertainties among the three investigators were resolved by three additional investigators (DA, MT, and GP), who were blinded to the study objectives.

For each eligible study, the following variables were systematically extracted: first author and year of publication, study type (in vitro or finite element analysis), type of fixed dental prosthesis (FDP), number of units, connector design variables (including cross-sectional area, height, width, geometry, or curvature), pontic span length, testing or analysis method (e.g., fracture load testing or stress analysis), and mechanical outcomes evaluated, including fracture resistance, fracture load, stress distribution, fatigue behavior, deformation/displacement, and failure mode.

When multiple experimental conditions or design configurations were assessed within a single study, only those relevant to the predefined variables of interest (connector design and pontic span length) were included in the analysis. Extracted data were subsequently organized and summarized in tabular form.

### 2.6. Risk of Bias Assessment

Risk of bias was independently assessed by two reviewers (LC and JMC. Any disagreements were resolved through discussion and, when necessary, by consultation with a third reviewer (AOB).

Given the absence of standardized and validated tools specifically designed for in vitro and FEA studies, a customized methodological quality assessment was performed based on previously reported criteria for laboratory and computational studies. The following domains were evaluated: (D1) specimen standardization, (D2) clarity of methodological description, (D3) reproducibility of experimental or computational procedures, (D4) outcome assessment, and (D5) statistical analysis.

Each domain was classified as low risk or unclear risk of bias. Overall, most included studies demonstrated a low risk of bias in specimen standardization and reproducibility, while some concerns were identified in the domains of methodological description and statistical analysis due to incomplete reporting. No study was classified as having a high risk of bias across the evaluated domains.

The results of the risk-of-bias assessment were summarized graphically to facilitate interpretation and comparison across studies [12].

### 2.7. Statistical Analysis

#### Qualitative Synthesis

A qualitative synthesis of all included studies was conducted, focusing on the main variables predefined in the eligibility criteria and extracted during the data collection process. Due to the methodological heterogeneity among studies—including differences in study design (in vitro vs. finite element analysis), prosthetic configurations, connector geometries, span lengths, and outcome assessment methods—a quantitative meta-analysis was not considered appropriate.

The qualitative analysis was structured around two main analytical components according to the objectives of the present review: (1) connector design variables, including cross-sectional area, height, width, geometry, and curvature; and (2) pontic span length.

For the connector design component, the synthesis explored variations across studies in terms of connector dimensions and geometry, as well as their influence on mechanical outcomes such as fracture resistance, fracture load, stress distribution, fatigue behavior, and failure mode. Particular attention was given to the consistency of findings regarding the relationship between connector size and mechanical performance, as well as to methodological differences in testing protocols and computational modeling.

For the pontic span length component, the qualitative analysis focused on the influence of span extension on biomechanical behavior, including stress concentration, bending moments, and structural stability. Differences in prosthesis configuration, number of units, and loading conditions were also considered as potential sources of variability across studies.

This structured qualitative synthesis allowed for the identification of consistent trends and methodological heterogeneity, providing a comprehensive framework for interpreting the biomechanical behavior of zirconia-based fixed dental prostheses.

## 3. Results

### 3.1. Study Selection

A total of 279 records were identified through database searching (PubMed, Scopus, Web of Science, and Embase). After removal of 72 duplicate records, 207 studies remained for screening. Of these, 170 were excluded based on title and abstract. A total of 37 full-text articles were assessed for eligibility, of which 26 were excluded due to lack of evaluation of connector- or span-related variables, inappropriate prosthesis design, irrelevant mechanical outcomes, or insufficient data. Finally, 11 studies were included in the qualitative synthesis. The study selection process is illustrated in Figure 1, following PRISMA 2020 recommendations [8].

### 3.2. Characteristics of Included Studies

The included studies comprised both in vitro and finite element investigations evaluating the effect of connector design and pontic span length on the mechanical behavior of zirconia FDPs. Most studies analyzed fracture resistance, fracture load, or stress distribution, and consistently identified the connector region as the main site of stress concentration and fracture initiation. The main characteristics and key findings of the included studies are summarized in Table 1, highlighting the consistent influence of these design variables on fracture resistance and stress distribution.

### 3.3. Main Findings

As detailed in Table 1, the included studies consistently demonstrated that connector design variables significantly influence the biomechanical behavior of zirconia FDPs. Increased connector dimensions were consistently associated with improved structural performance, particularly in terms of fracture resistance and reduction in stress concentration [21,22,23,24]. Conversely, longer pontic spans were associated with increased mechanical vulnerability due to higher bending moments and stress accumulation [25,26,27,28].

Several studies demonstrated that connector height may exert a greater influence on fracture resistance than connector width, while smoother geometrical transitions and increased cross-sectional area resulted in more favorable stress distribution patterns [29,30,31,32]. These findings confirm that connector design and pontic span length act as interdependent determinants of zirconia FDP biomechanics.

### 3.4. Risk of Bias

**Figure 2:**
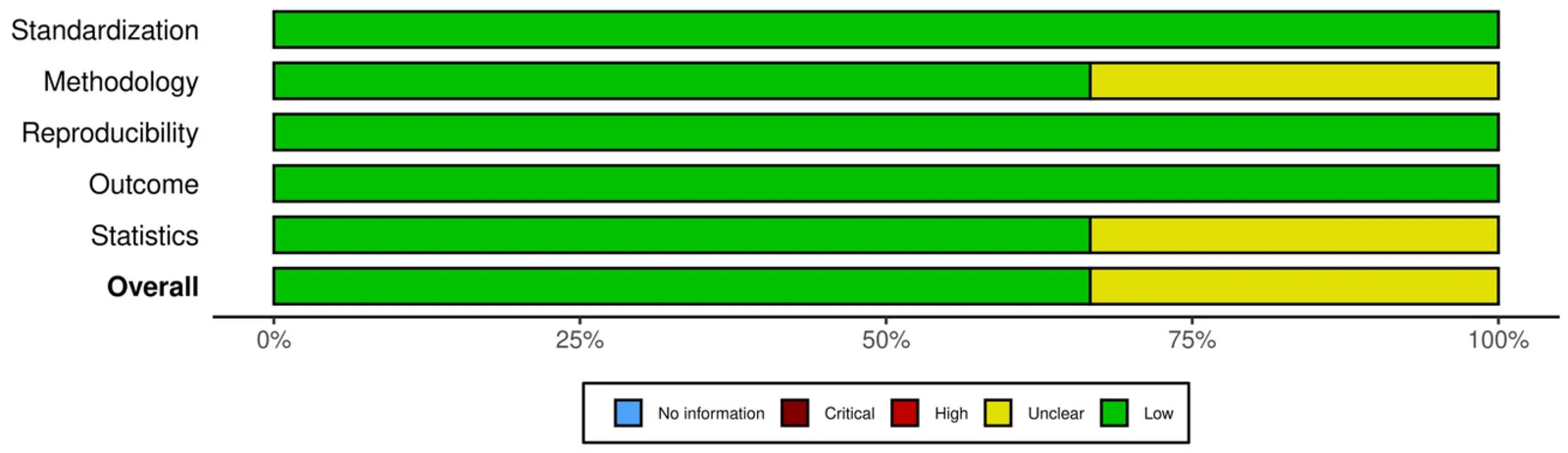
Summary of risk-of-bias assessment across all included studies according to each evaluated domain: specimen standardization (D1), methodological description (D2), reproducibility (D3), outcome assessment (D4), and statistical analysis (D5). Green indicates low risk of bias, and yellow indicates unclear risk of bias.

**Figure 3:**
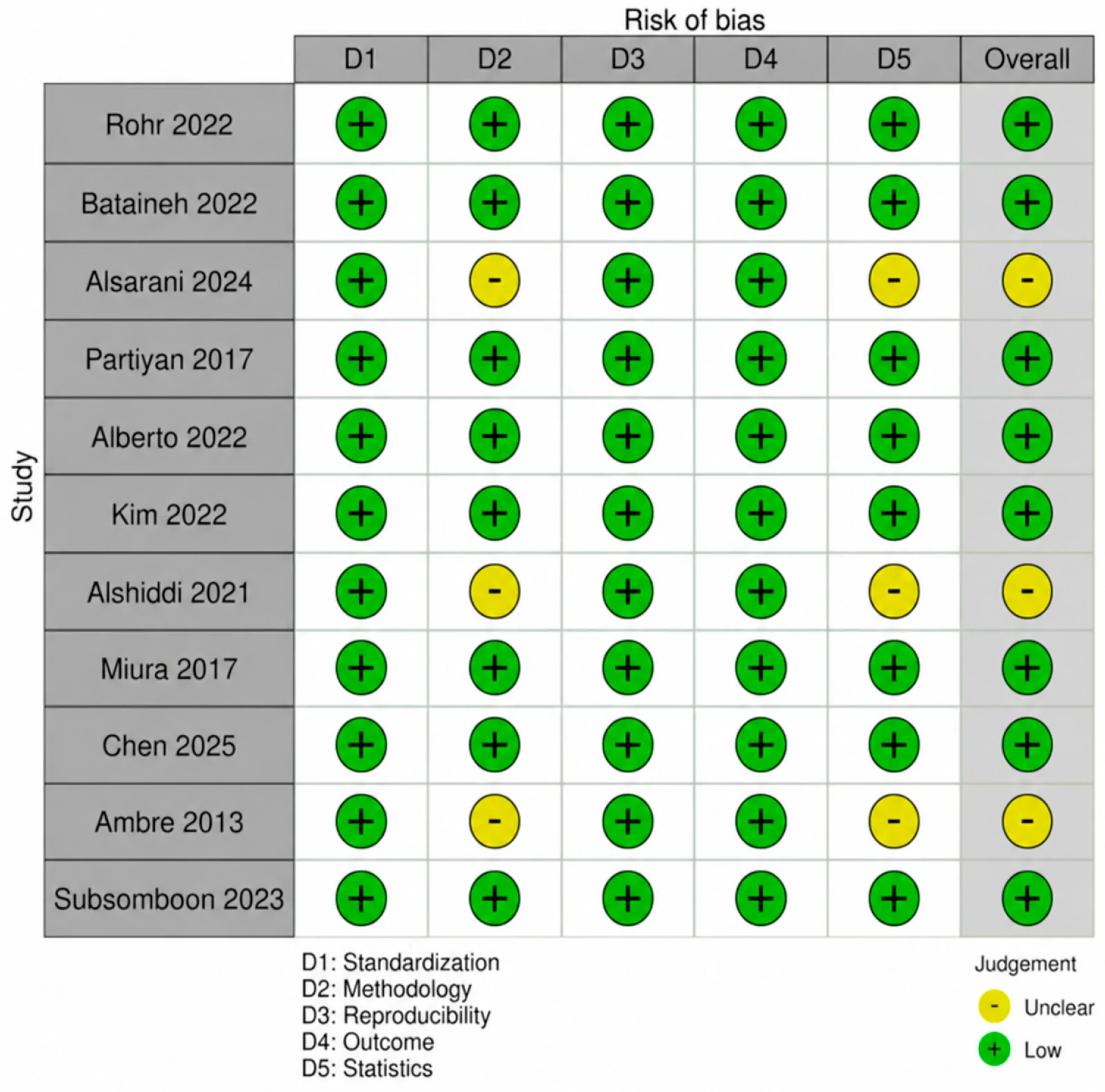
Risk-of-bias assessment of individual included studies across all evaluated domains: specimen standardization (D1), methodological description (D2), reproducibility (D3), outcome assessment (D4), and statistical analysis (D5). Green indicates low risk of bias, and yellow indicates unclear risk of bias [1,2,5,13,14,15,16,17,18,19,20].

The overall distribution of risk of bias across domains is presented in Figure 2, while the detailed assessment of individual studies is shown in Figure 3. In general, most included studies demonstrated a low risk of bias across the evaluated domains. Specifically, specimen standardization (D1) and reproducibility (D3) were consistently classified as low risk in all studies.

However, several studies exhibited an unclear risk of bias in the domains of methodological description (D2) and statistical analysis (D5), primarily due to insufficient reporting of experimental procedures or lack of detailed statistical information. In contrast, outcome assessment (D4) was generally well described across studies.

Notably, no study was classified as having a high risk of bias in any domain, indicating an overall acceptable methodological quality of the included studies. These visual representations enhance transparency and facilitate the interpretation of methodological quality across studies [11].

## 4. Discussion

The present systematic review demonstrated that the mechanical behavior of zirconia FDPs is strongly influenced by prosthetic design variables, particularly connector configuration and pontic span length. Although zirconia exhibits high strength and transformation-toughening mechanisms that contribute to crack resistance, the present findings indicate that material properties alone do not determine structural reliability. Instead, the way in which stresses are generated, transferred, and concentrated throughout the framework appears to play a decisive role in fracture development. This observation reinforces the concept proposed by Chevalier that the clinical performance of zirconia restorations is governed not only by material characteristics but also by the structural conditions under which mechanical loads are applied [33,34,35].

A consistent finding across the included studies was the identification of the connector region as the most mechanically vulnerable area of zirconia FDPs. However, the relevance of this observation goes beyond simply recognizing this region as a fracture-prone zone. Connectors act as the structural interface through which occlusal loads are transferred and redistributed between retainers and pontics, making them particularly sensitive to variations in both geometry and dimensions. Finite element analyses consistently reported peak tensile stresses in this region, while laboratory studies frequently identified connector-related failures as the predominant fracture pattern. From a biomechanical perspective, this behavior is expected, as connectors are simultaneously exposed to tensile, compressive, and bending forces during mastication. When local stresses exceed the fracture toughness of zirconia, crack initiation becomes more likely, particularly under cyclic loading conditions that may progressively weaken the ceramic structure. Although all included studies associated reduced connector dimensions with increased stress concentration, the magnitude of this effect varied according to framework configuration and analytical methodology. This variability suggests that connector performance cannot be explained solely by dimensional parameters, but should instead be interpreted within the broader biomechanical context of the entire restoration [14,17,36,37].

The available evidence consistently indicates that larger connector dimensions are associated with improved mechanical performance. Nevertheless, the relationship between connector size and fracture resistance is not strictly linear. While increases in connector height, width, thickness, or cross-sectional area generally reduce stress concentration, several studies suggest that each dimensional parameter may have a distinct contribution to structural stability. In particular, connector height appeared to exert a greater influence than width on resistance to flexural deformation. As reported by Kim et al., vertical enlargement produced a more pronounced improvement in load-bearing capacity, likely because of its effect on the moment of inertia of the framework. These findings are consistent with fundamental engineering principles, whereby increasing the vertical dimension of a structure produces a disproportionately greater enhancement in bending resistance than equivalent increases in width. However, the magnitude of the reported improvements varied among investigations, probably reflecting differences in prosthetic design, loading protocols, aging procedures, and zirconia compositions. Consequently, biomechanical optimization depends not only on increasing material volume but also on strategically modifying the structural geometry of the restoration [15,16,18,19,20].

Beyond connector dimensions, geometry also emerged as a critical determinant of biomechanical behavior. The reviewed evidence indicates that smoother transitions and larger curvature radii are associated with more favorable stress distribution patterns than designs with sharp internal angles. From a fracture mechanics perspective, abrupt geometric discontinuities act as stress concentrators, thereby facilitating crack initiation and accelerating crack propagation. Accordingly, mechanical performance depends not only on the amount of material present in the connector region, but also on how this material is distributed and integrated within the overall framework. Interestingly, some variability was observed among finite element studies regarding the exact location and magnitude of peak stress values. These differences may be attributed to variations in computational assumptions, mesh refinement strategies, loading configurations, and boundary conditions. Despite such methodological heterogeneity, the collective evidence consistently supports the concept that connector optimization should be approached as a three-dimensional design strategy rather than merely increasing connector size, particularly in clinical situations where anatomical limitations restrict dimensional enlargement [13,38,39].

The influence of pontic span length was equally relevant. Longer spans consistently generated less favorable biomechanical conditions, resulting in greater bending moments and higher tensile stress accumulation within the framework. Mechanically, increasing the distance between abutment teeth amplifies flexural deformation and increases the load transferred to the connector region. Consequently, even restorations fabricated from high-strength zirconia may become more susceptible to structural failure when span length exceeds biomechanically favorable limits. However, the effect of span extension was not uniform among studies. Differences in connector morphology, zirconia composition, loading protocols, and aging procedures likely contributed to variations in reported outcomes. Therefore, span length should not be regarded as an independent predictor of failure. Rather, its biomechanical impact appears to depend largely on how it interacts with connector design and framework geometry. This observation may explain why restorations with similar span lengths can exhibit markedly different mechanical behavior depending on their structural configuration [40,41,42].

The interaction between connector design and span length provides particularly important insight into the biomechanical behavior of zirconia FDPs. Several studies suggested that connector configurations capable of supporting functional loads in short-span restorations may not provide equivalent structural reliability when applied to longer prosthetic spans. This finding highlights the multifactorial nature of fracture development in zirconia restorations, where failure is often the result of cumulative stress concentration rather than the consequence of a single unfavorable design feature. Fatigue-related degradation likely plays a decisive role in this process, as repeated loading facilitates the slow growth of subcritical cracks until catastrophic failure occurs. Accordingly, the mechanical response of zirconia FDPs should be interpreted as the result of a dynamic interaction between material properties, connector configuration, and span extension rather than as the consequence of any individual parameter alone [43,44].

The overall methodological quality of the included studies strengthens the reliability of the observed biomechanical trends. Most investigations demonstrated low risk of bias regarding specimen standardization and reproducibility, whereas uncertainties were primarily related to methodological reporting and statistical analysis. Importantly, no study was classified as presenting a high risk of bias. Nevertheless, incomplete reporting within selected methodological domains may have contributed to some of the variability observed among investigations and should be considered when interpreting the results.

From a clinical perspective, these findings have direct implications for prosthetic planning. Connector design should be regarded as a primary biomechanical determinant rather than a secondary laboratory consideration. Whenever restorative space permits, increasing connector dimensions and avoiding abrupt geometric transitions may substantially reduce stress concentration and improve structural reliability. This consideration becomes particularly relevant in posterior restorations and extensive rehabilitations, where occlusal forces are greater and mechanical demands are inherently more challenging. Likewise, clinicians should recognize that long-span FDPs require more conservative biomechanical planning, as design configurations that perform satisfactorily in short-span restorations may become inadequate when functional demands increase. In this context, optimizing prosthetic design may be more effective for improving mechanical reliability than relying exclusively on materials with higher reported strength values.

Although the identified trends were consistent, their direct extrapolation to clinical practice should be approached with caution. The available evidence was derived exclusively from in vitro studies and FEA, both of which inevitably simplify the complexity of the oral environment. Variations in zirconia formulations, framework configurations, connector morphologies, loading conditions, aging protocols, and computational assumptions introduce substantial heterogeneity that limits direct comparison among studies. Furthermore, finite element models cannot fully reproduce biological variables such as periodontal ligament behavior, adaptive occlusal responses, moisture effects, or long-term degradation phenomena. Consequently, future research should prioritize methodological standardization and clinical validation to strengthen the translational applicability of biomechanical findings [45].

Nevertheless, the convergence of evidence obtained from both experimental and computational approaches strengthens confidence in the biomechanical trends identified in this review. Collectively, the available evidence indicates that connector dimensions, connector geometry, and pontic span length function as interdependent determinants of biomechanical performance rather than isolated design parameters. Their interaction governs stress distribution, crack initiation, fatigue progression, and structural reliability, ultimately influencing restoration longevity. Importantly, the present findings suggest that appropriate prosthetic design may exert a greater influence on long-term mechanical success than incremental improvements in zirconia material properties alone. Therefore, optimization of these variables should be considered a fundamental prerequisite for maximizing the predictability, durability, and biomechanical reliability of zirconia FDPs.

## 5. Strengths and Limitations

This systematic review integrates evidence from both in vitro and finite element studies, providing a comprehensive biomechanical perspective on zirconia FDP performance. A key strength lies in the focused evaluation of two clinically relevant variables: connector design and pontic span length.

However, several limitations must be acknowledged. First, the exclusive inclusion of laboratory and computational studies limits direct clinical extrapolation. Second, methodological heterogeneity across studies precluded quantitative synthesis. Third, variations in experimental conditions and model assumptions may have influenced the reported outcomes.

Rather than representing isolated observations, the consistency of these findings across both laboratory investigations and FEA suggests the existence of a common biomechanical pattern governing zirconia FDP behavior. This convergence strengthens the reliability of the available evidence and supports the concept that connector design should be considered a primary determinant of structural performance irrespective of the evaluation methodology employed.

## 6. Conclusions

Connector design and pontic span length are critical and interdependent determinants of the mechanical performance of zirconia fixed dental prostheses. Increased connector dimensions, particularly cross-sectional area and height, significantly improve fracture resistance and reduce stress concentration, while longer pontic spans negatively affect structural behavior. Optimization of these parameters is essential to enhance the biomechanical reliability and long-term clinical performance of zirconia FDPs. Future research should focus on standardizing experimental methodologies and incorporating clinical data to establish evidence-based prosthetic design guidelines.

The present review provides an integrated synthesis of experimental and computational evidence, highlighting consistent biomechanical trends across different methodologies and supporting the development of evidence-based design recommendations for zirconia FDPs.

## Figures and Tables

**Table 1:** Characteristics and main findings of included studies.

Author (Year)	Study Type	FDP Type	Units	Variable Analyzed	Method	Main Finding
Rohr et al. (2022) [1]	In vitro	Cantilever FDP	2-unit	Connector size	Fracture load	Connector size significantly influenced fracture resistance
Bataineh et al. (2022) [2]	FEA	3-unit FDP	3-unit	Connector size	Finite element analysis	Larger connectors improved fatigue resistance
Alsarani et al. (2024) [13]	In vitro + FEA	3-unit FDP	3-unit	Framework design/connector	Stress + fatigue	Highest stress was located at the connector region
Partiyan et al. (2017) [14]	In vitro	3-unit FDP	3-unit	Connector bulk	Fracture test	Increased connector thickness improved fracture resistance
Alberto et al. (2022) [15]	FEA	Implant-supported FDP	3-unit	Connector curvature	Stress distribution	Smaller radius increased stress concentration
Kim et al. (2022) [16]	In vitro	3-unit FDP	3-unit	Connector design	Fracture test	Connector height had greater influence than width
Alshiddi et al. (2021) [5]	In vitro	Cantilever FDP	2-unit	Span length + connector thickness	Fracture load	Longer spans reduced fracture resistance
Miura et al. (2017) [17]	FEA	Cantilever FDP	3-unit	Framework design	Stress analysis	Increased framework dimensions reduced stress
Chen et al. (2025) [18]	FEA	3-unit FDP	3-unit	Connector cross-sectional area	Stress analysis	Larger CSA reduced stress concentration
Ambré et al. (2013) [19]	In vitro	3-unit FDP	3-unit	Connector dimension	Fracture load	Fracture strength depended primarily on connector size
Subsomboon et al. (2023) [20]	In vitro	3-unit FDP	3-unit	Connector configuration	Fracture load	Cross-sectional area was more relevant than shape

## Data Availability

The raw data supporting the conclusions of this article will be made available by the authors without undue reservation.

## Supplementary Materials

The following supporting information can be downloaded at: https://www.mdpi.com/article/10.3390/dj14080500/s1, File S1: PRISMA 2020 checklist.
